# Impact of *Helicoverpa zea* (Lepidoptera: Noctuidae) feeding on yield components in double-cropped soybean with determinate and indeterminate growth habits

**DOI:** 10.1093/jee/toaf211

**Published:** 2025-08-23

**Authors:** Taynara Possebom, Dominic Reisig, Anders Huseth, Rachel Vann

**Affiliations:** Department of Entomology and Plant Pathology, North Carolina State University, Raleigh, NC, USA; Department of Entomology and Plant Pathology, North Carolina State University, Vernon G. James Research and Extension Center, Plymouth, NC, USA; Department of Entomology and Plant Pathology, North Carolina Plant Science Institute, North Carolina State University, Raleigh, NC, USA; Department of Crop and Soil Sciences, North Carolina Plant Sciences Initiative, North Carolina State University, Raleigh, NC, USA

**Keywords:** corn earworm, relative maturity, threshold, row crop

## Abstract

*Helicoverpa zea* (Boddie) (Lepidoptera: Noctuidae) injury to soybean (*Glycine max* L. Merrill) may result in yield loss. To minimize loss, economic thresholds are used to make treatment decisions. However, many of these thresholds are based on studies using determinate soybean varieties, and it remains unclear whether indeterminate varieties have a greater capacity to compensate for *H. zea* herbivory compared to determinate ones. For this reason, we investigated whether soybean varieties with different growth habits vary in their ability to compensate for *H. zea* feeding injury in double-crop systems. We hypothesized that yield and yield components would not differ by growth habit (indeterminate and determinate) or relative maturity (5.2 and 5.4/5.5), regardless of *H. zea* pressure. We conducted experiments across multiple locations over 2 yr, using soybean varieties with different growth habits but similar relative maturities. We found a significant relationship between larval infestation and growth habit, specifically, for individual seed weight, with indeterminate varieties showing increased seed weight as larval infestation increased. However, we did not observe this relationship for other yield components or overall yield. Our findings suggest that indeterminate soybean varieties may have greater compensatory ability in response to biotic stressors than determinate varieties, likely due to their extended vegetative growth window may allow for greater compensation. Nevertheless, we conclude that current economic thresholds remain appropriate for both determinate and indeterminate soybean varieties in double-crop systems. We recommend additional research across more varieties and environments with higher *H. zea* pressure to better understand soybean compensatory responses to insect injury and damage.

## Introduction

*Helicoverpa zea* (Boddie) (Lepidoptera: Noctuidae), commonly known as corn earworm or bollworm, is polyphagous and feeds on over 100 plant species. This pest can cause economic injury and damage in common field crops such as corn (*Zea mays* L.), cotton (*Gossypium hirsutum* L.), and soybean (*Glycine max* (L.) Merrill) (Neunzig 1969). In soybean, *H. zea* is one of the costliest pests (Musser et al. 2021, Musser et al. 2022) and can cause direct yield loss by feeding on flowers and fruits, and can cause indirect yield loss by feeding on leaves (Fitt 1989, Eckel et al. 1992a, Reisig et al. 2017).

Soybean varieties are classified based on maturity group and morphological growth habit, which can be determinate or indeterminate (Fehr et al. 1977). Determinate soybean varieties terminate vegetative growth on the mainstem when flowering begins, or shortly thereafter, while indeterminate soybean varieties continue vegetative growth on the mainstem after flowering begins through continued stem elongation and leaf production (Robinson and Wilcox 1998). Historically, soybean varieties with a determinate growth habit were widely produced in the southeastern United States. While determinate types have historically dominated production in the southeastern United States, indeterminate varieties are increasingly planted, even in double-crop systems behind small grains or corn, due to perceived yield advantages (Boote 1981, Savoy et al. 1992, Heatherly 1996, Vann et al. 2021). Double-cropping can enhance per-acre returns and reduce risk by diversifying crops within the same season. In North Carolina, the recommended economic threshold for *H. zea* is one larva per row foot using a beat cloth sampling method, or 3.1 larvae per 15 sweeps, based on determinate growth habit. (Herbert et al. 2009). Thresholds used throughout the United States are also based on determinate varieties (McAlister and Krober 1958, Hicks et al. 1969, Kincade et al. 1971, Smith and Bass 1972a, Hammond and Pedigo 1982). A single threshold study for *H. zea* in soybean focused on indeterminate growth habits (Adams et al. 2016), but it was conducted in the Midsouth United States under artificial infestation, limiting broader applicability.

Previous research in full-season soybean (planted late April to May) found no yield differences between soybean growth habits when exposed to varying levels of *H. zea* pressure (Schug et al. 2022), suggesting current thresholds may be broadly applicable. However, more work is needed across a wider range of varieties and planting dates. For example, there is limited information on how soybean varieties with different growth habits compensate for *H. zea* feeding when planted in double-crop conditions (planted mid-June or later) (Egli and Cornelius 2009). This is important because *H. zea* populations tend to be higher in double-cropped soybean (Bradley et al. 1986), increasing the risk of economic damage.

Planting date and maturity group may also interact to influence *H. zea* feeding patterns in the field; neonates are commonly found in young leaves and flowers, while older instars are found most often on flowers and pods (McWilliams 1983, Eckel et al. 1992b). Under laboratory conditions, second instars preferentially feed on leaves and small pods when given a choice, while fourth instars preferentially feed on pods with fully developed seed (Suits et al. 2017). Although this suggests tissue-specific feeding, field data show second and older instars are evenly distributed in the canopy (Reisig et al. 2021). Indeterminate soybean may produce a succession of pods at various stages, potentially supporting higher *H. zea* densities (Terry et al. 1989, Suits et al. 2017). However, there is no evidence of feeding preference differences between growth habits (Reisig et al. 2020).

Growth habit may also influence a plant’s ability to compensate for yield loss, depending on tissue availability at the time of infestation. For example, *H. zea* moths prefer to lay eggs in flowering-stage soybean, and this preference is independent of the growth habit (Possebom et al. 2024). However, at full bloom, an indeterminate plant may already have small pods on the lower nodes, whereas a determinate plant typically has only flowers at that stage. While not observed in full-season soybean (Schug et al. 2022), compensation capacity in double-crop soybean remains poorly understood. Flower loss is often considered low-risk due to compensatory potential (Kincade et al. 1971, Smith and Bass 1972b, Reisig et al. 2017). However, when the plant enters the podding stages, the probability of yield loss is higher as the plant requires a significant amount of water and nutrients during these stages to produce seed (McAlister and Krober 1958, Smith and Bass 1972a). Determinate types may struggle to compensate under stress (McPherson and Moss 1989), whereas indeterminate types might recover by producing additional pods; however, this remains untested.

The primary objective of our study was to investigate whether soybean varieties with a determinate or indeterminate growth habit planted in a double-cropped timing compensated differently to *H. zea* feeding across environments with different population levels. We hypothesized that indeterminate and determinate soybean growth habit would not differ by yield and yield components across *H. zea* natural infestation.

The secondary objective of our study was to investigate if soybean varieties with a relative maturity of 5.2 and 5.4/5.5 planted in a double-cropped timing also compensated differently to *H. zea* feeding across environments with different population levels. We hypothesized that soybean relative maturities (5.2, 5.4/5.5) would not differ by yield and yield components across *H. zea* natural infestation.

To test both hypotheses, we planted replicated small-plot field experiments with paired varieties sharing the same relative maturity (5.2 and 5.4/5.5), but differing in growth habit (determinate and indeterminate). We planted these experiments in different environments (location and year) that experienced varying levels of *H. zea* infestation. We measured larval infestation levels throughout the season and collected yield component information. We evaluated differences in yield components between indeterminate and determinate varieties within the same maturity group, hypothesizing that indeterminate types may exhibit better compensation capability from *H. zea* herbivory.

## Materials and Methods

### Experimental Design, Planting, and Plot Maintenance

We planted soybean field trials during mid-June with small plot research planter (SRES—Classic Aire Research Planter; Kincaid Equipment Manufacturing, Haven, Kansas, United States) at 5 locations in North Carolina (Table 1) (USDA 2023). While we planted at the Tidewater Research Station in Plymouth and the Upper Coastal Plains Research Station in Rocky Mount in both years, we used different fields each year. We followed production recommendations by North Carolina Cooperative Extension (Hardy et al. 2009, Stowe et al. 2018).

**Table 1.** Double-cropped planting dates across multiple locations in North Carolina

Year	Field locations	County	Soil type	Planting date
2021	Central Crops Research Station	Wake	Norfolk loamy sand, 2% to 6% percent slopes (NoB)	14 June
	Tidewater Research Station	Washington	Portsmouth fine sandy loam (Pt)	15 June
	Upper coastal plains	Edgecombe	Goldsboro fine sandy loam, 0% to 2% slopes (GoA)	15 June
	Research station			
	Roper farm area	Washington	Arapahoe fine sandy loam (Ap)	17 June
2022	Tidewater Research Station	Washington	Cape Fear loam, 0% to 2% slopes, rarely flooded (Cf)	6 June
	Upper coastal plains	Edgecombe	Goldsboro fine sandy loam, 0% to 2% slopes (GoA)	6 July^a^
	Research station			
	Pantego farm area	Beaufort	Arapahoe fine sandy loam (Ap)	7 June

a This location was first planted on June 7, but due to poor emergence, we replanted in July.

We arranged varieties in a randomized complete block design with four replications at each environment. Each plot measured 12.19 m in length and eight rows in width, with 91 cm spacing between rows. In each block, our treatments were arranged in a 2 × 2 factorial design with 2 paired soybean varieties that shared similar relative maturity but had different growth habits. The first pair consisted of relative maturity 5.2; one variety had an indeterminate growth habit (AG52XF0 XF/SR; Bayer Crop Science, St Louis, Missouri, United States), while the other had a determinate growth habit (5220R2X/SR RR2X/SR; Bayer Crop Science). The second pair had a variety with a relative maturity of 5.4 and an indeterminate growth habit (AG54XF0 XF/SR; Bayer Crop Science), while the other had a variety with a relative maturity of 5.5 and a determinate growth habit (AG55XF0 XF; Bayer Crop Science). All soybean varieties were tolerant to dicamba, glyphosate, and glufosinate.

In all environments, we applied a pre-emergent herbicide, S-metolachlor and fomesafen (1.067 g a.i./ha) of Prefix, Syngenta, Greensboro, North Carolina, Untied States. A month after soybean emergence, we applied post-emergent herbicides, glyphosate (897 g a.i./ha of Roundup, Bayer Crop Science), and cloransulam-methyl (176 g a.i./ha of FirstRate, Corteva Agriscience, Indianapolis, Indiana, United States). Because weed densities were assessed visually and we still observed high weed pressure at the Beaufort County location after the first post-emergent application compared to other locations, we made an additional post-emergent application of glyphosate (897 g a.i./ha; Roundup, Bayer Crop Science) and flumiclorac-pentyl ester (292 g a.i./ha; Resource, Valent USA Corporation, Cary, North Carolina, United States).

We applied herbicides using a mechanical sprayer (Lee Avenger sprayer, LeeAgra, Lubbock, Texas, United States) calibrated to deliver 187.08 liter/ha at 6.43 km/h and 206.84 kPa using a hollow cone with a TP8003 flat fan nozzle (TeeJet, Glendale Heights, Illinois, United States). The Edgecombe County location in 2022 received a late-season herbicide application of paraquat dichloride (174 g a.i./ha of Gramoxone, Syngenta) sprayed with a CO_2_-powered backpack sprayer calibrated to deliver 93.5 liter/ha at 4 km/h and 128.67 kPa with TX10 hollow cone spraying nozzles (TeeJet).

During 2021, kudzu bug (*Megacopta cribraria* Fabricius) infested both growth habits in the Wake County location during soybean stage V7-V8, which is the period when the soybean plant begins to shift from vegetative to reproductive. The Edgecombe County location also had a *M. cribraria* and bean leaf beetle (*Cerotoma trifurcata* Forster) infestation during the R7-R8 stages, which is the initiation of maturity. To limit any confounding effects from secondary pests, we applied bifenthrin (154 g a.i./ha of Quali-Pro Bifenthrin, Makhteshim Agan of North America, ADAMA, Raleigh, North Carolina, United States) using the same conditions as mentioned above.

### Data Collection

Once the soybean plants began blooming, we conducted weekly sampling in each plot to record the number of *H. zea* using a 0.42 m^2^ (0.71 m in length × 0.59 m in width) beat cloth (sampling method design by Bignell 1875 (Barnard 2018)). Plants were vigorously agitated onto the cloth for about 10 s, and the number of *H. zea* larvae was recorded. Each independent sample totaled 1.42 m of soybean row. We took 2 subsamples from the middle 2 rows in each plot; each subsample was separated by a 4 m distance down the row. The 2 subsamples were summed to generate a plot-level count of *H. zea* larvae across 2.8 m of linear row per plot. We collected samples when 95% of all plots were at the R1 stage, one open flower at any node on the main stem, until R6, full seed stage (Pedersen et al. 2018). We sampled 7 wk at each location and calculated the total number of larvae per square meter (larvae/m^2^). This was accomplished by summing the total number of larvae collected throughout the season and dividing by 0.84 m^2^, as we sampled each plot twice using the beat cloth method described above.

When 95% of soybean plants in the plots reached full maturity (R8), we destructively sampled and counted all plants in a randomly selected one-linear-meter (LM) section of a single row to determine soybean yield components, including the total number of pods, total number of seeds, and total seed weight. We threshed the hand-harvested plants in 18.9 liter buckets to catch pods and loose seeds while removing the stems and other unwanted material. Next, we labeled the samples and placed them into paper envelopes to bring them to the laboratory. We counted on our hands the total number of pods for each plot. We also counted the total number of seeds using an automatic seed counter (Old Mill Seed Counter Model 850-3. International Marketing and Design Corporation, IMD, San Antonio, Texas, United States). We dried the seeds in an oven (Thermo Scientific precision compact mechanical, Fisher Scientific, Waltham, Massachusetts, United States) at 65 °C for 72 h and then weighed them to determine the total dry seed weight.

We then calculated the total number of pods per plant (pods per LM/plants per LM), total number of seeds per plant (seeds per LM/plants per LM), total number of seeds per pods (seeds per LM/pods per LM), and individual seed weight (seed weight in g per LM/seeds per LM). To determine plot-level yield (kg/ha), we harvested the middle 2 rows of each plot using a plot combine (Model 8-XP, Kincaid equipment manufacturing, Haven, Kansas, United States) except at the Pantego farm area (Beaufort County, 2022) because it was accidentally harvested by the grower. After harvest, we converted soybean yield to 13% moisture content, and we took seed subsample from each plot.

### Statistical Analysis

We conducted an analysis of covariance using linear mixed models for all 7 locations, performing individual analyses for each of our dependent variables: total number of pods (pods/LM), total number of seeds (seeds/LM), average number of pods per plant, average number of seeds per plant, average number of seeds per pod, individual seed weight (g), seed weight (g/LM), and plot-level average yield (kg/ha). The covariate was the total number of *H. zea* larvae in each plot throughout the season (infestation/m^2^). The fixed effects were growth habit (determinate and indeterminate), relative maturity (5.2 and 5.4/5.5). The random effects were location nested within year (year: location) and block nested within location nested within year (year: location: block). We investigated all the interactions of categorical growth habit or variety predictors with the *H. zea* infestation covariate.

We generated all statistical analyses with RStudio version 1.2.5042 (RStudio Core Team 2020). To fit the analysis of variance, we used the anova function Type III from the *car* package (Fox and Weisberg 2019). To fit the linear mixed models, we used the lmer function from the *lme4* package (Pinheiro and Bates 2000, Andrade and Estévez-Pérez 2014, Bates et al. 2015). We performed Tukey honestly significant difference (HSD) test at a significance level of 5% (α = 0.05) on the highest order interaction that was significant. In the case that no interactions were significant, we report the mean separations for the significant main effects. Tukey adjustments were replaced with Sidak when necessary to accommodate multiple sets of pairwise comparisons, using the *multcomp* and *glht* packages (Hothorn et al. 2008).

## Results

Our results are divided into yield components (total number of pods, total number of seeds, average number of pods per plant, average number of seeds per plant, average number of seeds per pod, and total seed weight) and yield. If there was an interaction, we focused on the highest-order significant interaction. If not, we focused on the significant main effects.

### Total Number of Pods (Pods/LM)

The 4-way interaction for the total number of pods was not significant: larval infestation × growth habit × relative maturity × year (*F*_1,76_ = 0.4954, *P *= 0.4836). Similarly, none of the 3-way interactions were statistically significant: larval infestation × growth habit × relative maturity (*F*_1,76_ = 0.9294, *P *= 0.3380); growth habit × relative maturity × year (*F*_1,73_ = 3.9239, *P *= 0.052); larval infestation × growth habit × year (*F*_1,73_ = 0.1739, *P *= 0.6778), or larval infestation × relative maturity × year (*F*_1,73_ = 1.5176, *P *= 0.2218).

**Fig. 1.**
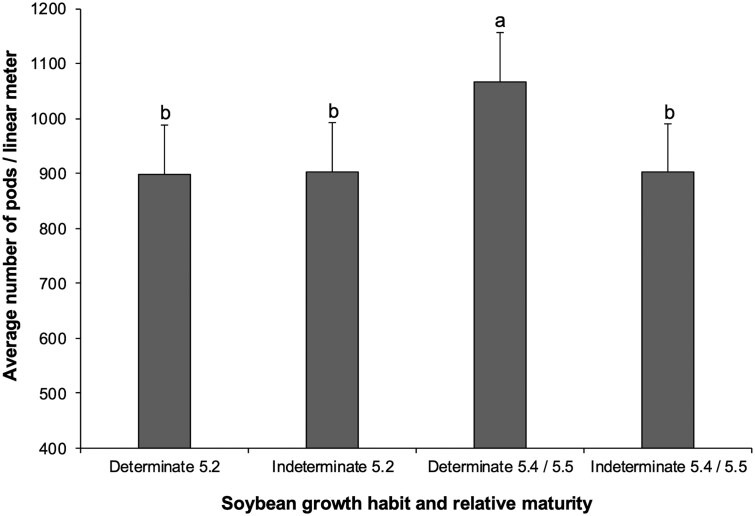
Two-way interaction between soybean growth habit (determinate and indeterminate) and relative maturity (5.2, 5.4/5.5) for the average number of pods per linear meter across the season. Means (±SE) sharing the same letter do not differ significantly (α = 0.05; Sidak-adjusted pairwise comparisons of estimated marginal means).

The only significant 2-way interaction was growth habit × relative maturity (*F*_1,73_ = 7.5632, *P *= 0.007; Fig. 1). All other 2-way interactions not significant: larval infestation × year (*F*_1,65_ = 0.7676, *P *= 0.3841), larval infestation × growth habit (*F*_1,73_ = 0.0219, *P *= 0.8826); larval infestation × relative maturity (*F*_1,74_ = 2.0236, *P *= 0.1590), growth habit × year (*F*_1,72_ = 0.1022, *P *= 0.7501), and relative maturity × year (*F*_1,72_ = 0.3003, *P *= 0.5854).

The main effects of larval infestation (*F*_1,65_ = 0.1265, *P *= 0.7232) and year (*F*_1,5_ = 0.4629, *P *= 0.5229) were not significant.

### Total Number of Seeds (Seeds/LM)

The 4-way interaction for the total number of seeds was not significant: larval infestation × growth habit × relative maturity × year (*F*_1,77_ = 0.4522, *P *= 0.5033). Similarly, none of the 3-way interactions were statistically significant: larval infestation × growth habit × relative maturity (*F*_1,77_ = 2.0588, *P *= 0.1553); growth habit × relative maturity × year (*F*_1,73_ = 1.2698, *P *= 0.2634); larval infestation × growth habit × year (*F*_1,74_ = 0.3037, *P *= 0.5832), or larval infestation × relative maturity × year (*F*_1,74_ = 1.2162, *P *= 0.2736).

All 2-way interactions were not significant: growth habit × relative maturity (*F*_1,73_ = 2.1700, *P *= 0.1449), larval infestation × year (*F*_1,65_ = 3.0018, *P *= 0.0895), larval infestation × growth habit (*F*_1,74_ = 1.6135, *P *= 0.2079), larval infestation × relative maturity (*F*_1,74_ = 0.0370, *P *= 0.8479), growth habit × year (*F*_1,72_ = 0.0035, *P *= 0.9527), and relative maturity × year (*F*_1,72_ = 1.2021, *P *= 0.2765).

The main effects of growth habit (*F*_1,72_ = 0.0003, *P *= 0.9859), relative maturity (*F*_1,72_ = 0.0505, *P *= 0.8228), larvae infestation (*F*_1,65_ = 1.3728, *P *= 0.2471), and year (*F*_1,6_ = 0.5026, *P *= 0.5054) were not significant.

### Average Number of Pods Per Plant

**Fig. 2.**
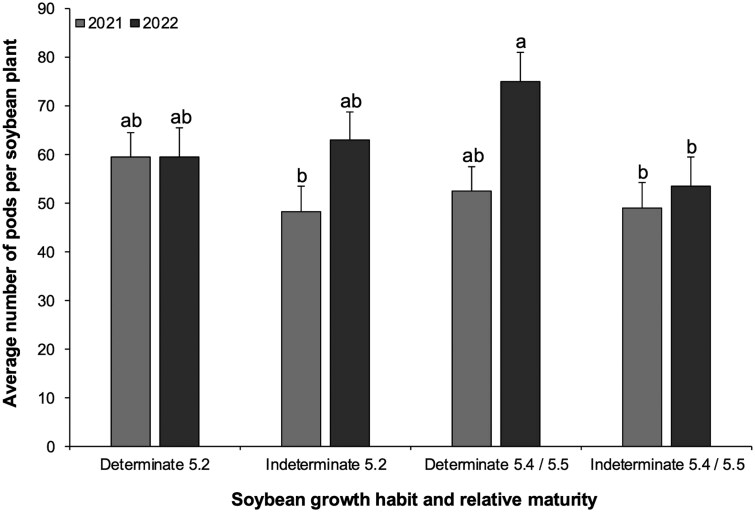
Three-way interaction among soybean growth habit, relative maturity, and year for the average number of pods per soybean plant across the season. Means (±SE) sharing the same letter do not differ significantly (α = 0.05; Sidak-adjusted pairwise comparisons of estimated marginal means).

The 4-way interaction for average number of pods per plant was not significant: larval infestation × growth habit × relative maturity × year (*F*_1,76_ = 0.9169, *P *= 0.3413). The only significant 3-way interaction was growth habit × relative maturity × year (*F*_1,73_ = 10.7698, *P *= 0.0015; Fig. 2). All other two-way interactions not significant: larval infestation × growth habit × relative maturity (*F*_1,76_ = 0.1971, *P *= 0.6583); larval infestation × growth habit × year (*F*_1,74_ = 0.6554, *P *= 0.4220), or larval infestation × relative maturity × year (*F*_1,74_ = 12.0249, *P *= 0.1589).

The only significant 2-way interaction was relative maturity × year (*F*_1,72_ = 4.0198, *P *= 0.048). All other 2-way interactions not significant: growth habit × relative maturity (*F*_1,73_ = 2.8054, *P *= 0.0982); larval infestation × year (*F*_1,69_ = 0.0963, *P *= 0.7572); larval infestation × growth habit (*F*_1,74_ = 0.0028, *P *= 0.8672); larval infestation × relative maturity (*F*_1,74_ = 2.4317, *P *= 0.1231); and growth habit × year (*F*_1,72_ = 0.7376, *P *= 0.3932).

The main effects of larvae infestation (*F*_1,69_ = 0.3744, *P *= 0.5426) and year (*F*_1,7_ = 2.2946, *P *= 0.1722) were not significant.

### Average Number of Seeds per Plant

The 4-way interaction for average number of seeds per plant was not significant: larval infestation × growth habit × relative maturity × year (*F*_1,77_ = 1.0055, *P *= 0.3191).

**Fig. 3.**
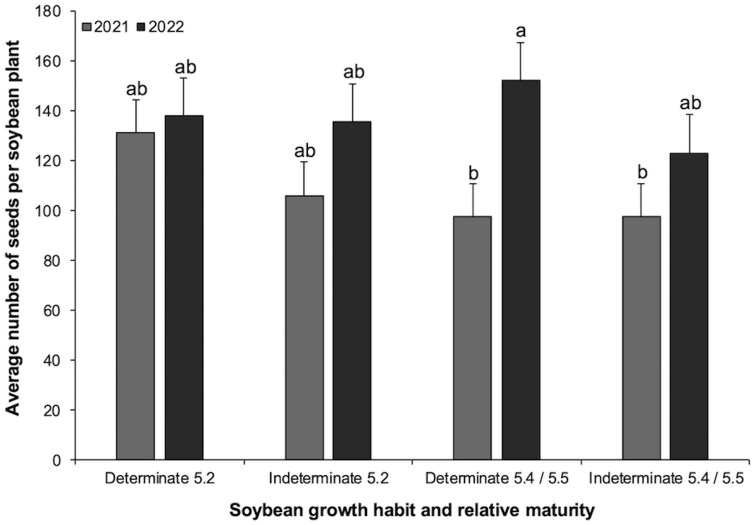
Three-way interaction among soybean growth habit, relative maturity, and year for the average number of seeds per soybean plant across the season. Means (±SE) sharing the same letter do not differ significantly (α = 0.05; Sidak-adjusted pairwise comparisons of estimated marginal means).

The only significant three-way interaction was growth habit × relative maturity × year (*F*_1,74_ = 5.4567, *P *= 0.0222; Fig. 3). All other 2-way interactions not significant: larval infestation × growth habit × relative maturity (*F*_1,77_ = 0.6848, *P *= 0.4104); larval infestation × growth habit × year (*F*_1,75_ = 0.0874, *P *= 0.7683), or larval infestation × relative maturity × year (*F*_1,75_ = 1.5110, *P *= 0.2228).

The only significant 2-way interaction was relative maturity × year (*F*_1,72_ = 5.0086, *P *= 0.0282). All other 2-way interactions not significant: growth habit × relative maturity (*F*_1,74_ = 0.3979, *P *= 0.5301); larval infestation × year (*F*_1,57_ = 0.4278, *P *= 0.5156); larval infestation × growth habit (*F*_1,75_ = 0.6885, *P *= 0.4093); larval infestation × relative maturity (*F*_1,75_ = 0.2027, *P *= 0.6538); and growth habit × year (*F*_1,72_ = 0.1649, *P *= 0.6858).

The main effects of larvae infestation (*F*_1,57_ = 1.7937, *P *= 0.1857) and year (*F*_1,6_ = 1.5422, *P *= 0.2548) were not significant.

### Average Number of Seeds per Pod

The 4-way interaction for average number of seeds per pod was not significant: larval infestation × growth habit × relative maturity × year (*F*_1,92_ = 0.0339, *P *= 0.8542). Similarly, none of the 3-way interactions were statistically significant: larval infestation × growth habit × relative maturity (*F*_1,92_ = 0.3770, *P *= 0.5407), growth habit × relative maturity × year (*F*_1,92_ = 0.5063, *P *= 0.4785), larval infestation × growth habit × year (*F*_1,92_ = 0.0071, *P *= 0.9331), or larval infestation × relative maturity × year (*F*_1,91_ = 0.0456, *P *= 0.8314).

All 2-way interactions were not significant: growth habit × relative maturity (*F*_1,92_ = 0.0368, *P *= 0.8482), larval infestation × year (*F*_1,96_ = 0.5249, *P *= 0.4705), larval infestation × growth habit (*F*_1,92_ = 1.6994, *P *= 0.1956), larval infestation × relative maturity (*F*_1,91_ = 1.3390, *P *= 0.2502), growth habit × year (*F*_1,91_ = 0.0028, *P *= 0.9578), and relative maturity × year (*F*_1,91_ = 0.8323, *P *= 0.3640).

**Fig. 4.**
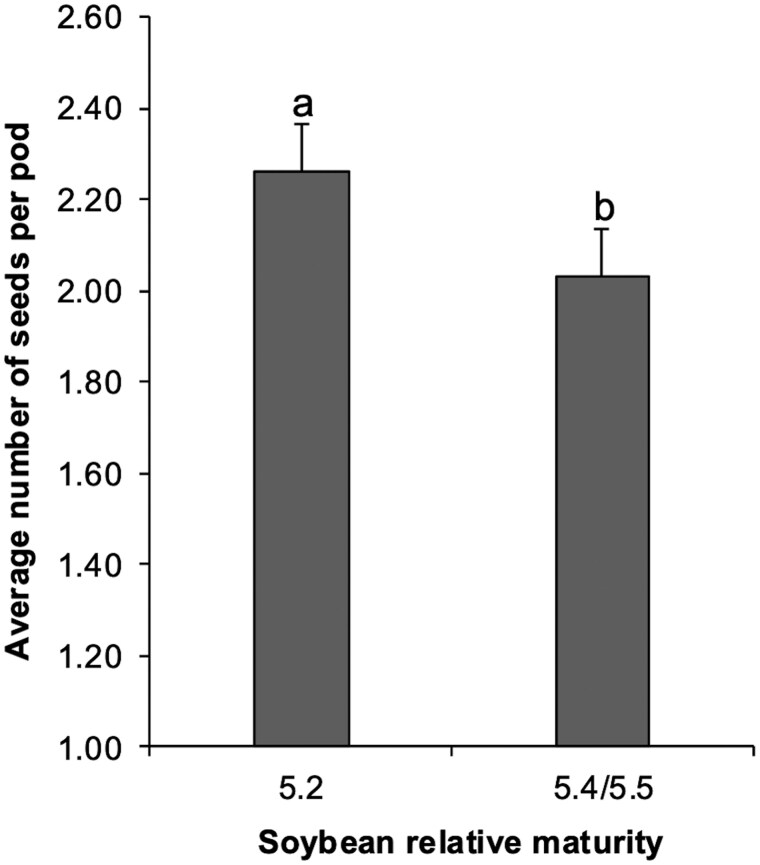
Average number seeds per pod for each relative maturity (5.2, 5.4/5.5) across the season. Means (±SE) followed by the same letter are not significantly different using Tukey’s HSD test at α = 0.05.

The main effects of relative maturity (*F*_1,91_ = 5.2508, *P *= 0.0242) were significant (Fig. 4). While growth habit (*F*_1,91_ = 2.5539, *P *= 0.1134), larvae infestation (*F*_1,96_ = 1.6547, *P *= 0.2014), and year (*F*_1,7_ = 0.1405, *P *= 0.7181) were not significant.

### Individual Seed Weight (g)

The 4-way interaction for individual seed weight was not significant: larval infestation × growth habit × relative maturity × year (*F*_1,92_ = 0.7412, *P *= 0.3915). Similarly, none of the 3-way interactions were statistically significant: larval infestation × growth habit × relative maturity (*F*_1,91_ = 2.1113, *P *= 0.1496); growth habit × relative maturity × year (*F*_1,91_ = 1.2045, *P *= 0.2753); larval infestation × growth habit × year (*F*_1,91_ = 0.5444, *P *= 0.4625), or larval infestation × relative maturity × year (*F*_1,91_ = 0.0791, *P *= 0.7791).

**Fig. 5.**
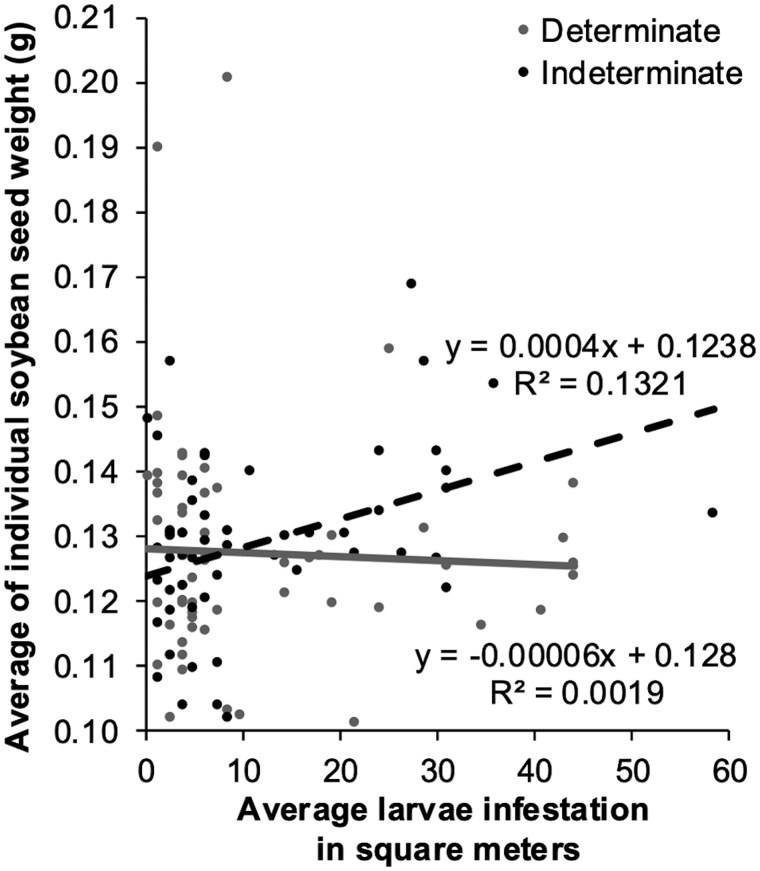
Interaction between soybean growth habit and average *H. zea* larval infestation (m^2^) across the season for the average individual soybean seed weight (g).

The only significant 2-way interaction was larval infestation × growth habit (*F*_1,91_ = 4.7550, *P *= 0.0317, Fig. 5), where indeterminate growth habit showed a moderate positive correlation (*R*^2^ = 0.1321) between average of individual seed weight (g) and average *H. zea* larvae infestation (m^2^), indicating as larvae infestation increases individual seed weight also increases. However, determinate growth habits showed almost no correlation (*R*^2^ = 0.0019), suggesting larvae infestation had a minimal impact on individual seed weight for determinate growth habit (Fig. 4).

All other 2-way interactions not significant: growth habit × relative maturity (*F*_1,91_ = 1.1772, *P *= 0.2807), larval infestation × year (*F*_1,95_ = 0.1825, *P *= 0.6701), larval infestation × relative maturity (*F*_1,91_ = 0.1229, *P *= 0.7266), growth habit × year (*F*_1,90_ = 0.7751, *P *= 0.3809), and relative maturity × year (*F*_1,90_ = 0.1863, *P *= 0.6670).

The main effects of growth habit (*F*_1,90_ = 1.3446, *P *= 0.2492), relative maturity (*F*_1,90_ = 1.0125, *P *= 0.3169), larvae infestation (*F*_1,95_ = 1.0291, *P *= 0.3129), and year (*F*_1,6_ = 0.0624, *P *= 0.8107) were not significant.

### Seed Weight (g/LM)

The 4-way interaction for seed weight was not significant: larval infestation × growth habit × relative maturity × year (*F*_1,75_ = 0.9488, *P *= 0.3331). Similarly, none of the 3-way interactions were statistically significant: larval infestation × growth habit × relative maturity (*F*_1,75_ = 0.8511, *P *= 0.3591); growth habit × relative maturity × year (*F*_1,72_ = 3.0474, *P *= 0.085); larval infestation × growth habit × year (*F*_1,72_ = 0.0138, *P *= 0.9067), or larval infestation × relative maturity × year (*F*_1,73_ = 1.4938, *P *= 0.2255).

The only significant 2-way interaction was larval infestation × year (*F*_1,91_ = 4, *F*_1,60_ = 5.6336, *P *= 0.0208). However, both years showed no correlation (2021: *R*^2^ = 0.0084, 2022: *R*^2^ = 0.0019), suggesting larvae infestation has no impact on seed weight (g/LM) by year.

All other 2-way interactions not significant: growth habit × relative maturity (*F*_1,72_ = 1.2633, *P *= 0.2647), larvae infestation × growth habit (*F*_1,73_ = 0.0270, *P *= 0.8700), larval infestation × relative maturity (*F*_1,73_ = 0.2643, *P *= 0.6087), growth habit × year (*F*_1,71_ = 0.6028, *P *= 0.4400), and relative maturity × year (*F*_1,71_ = 1.1081, *P *= 0.2960).

The main effects of growth habit (*F*_1,71_ = 0.5891, *P *= 0.4453), relative maturity (*F*_1,71_ = 1.8942, *P *= 0.1730), larvae infestation (*F*_1,60_ = 0.6020, *P *= 0.4408), and year (*F*_1,6_ = 0.3571, *P *= 0.5729) were not significant.

### Yield (Kg/ha)

The 4-way interaction for yield was not significant: larval infestation × growth habit × relative maturity × year (*F*_1,62_ = 0.0335, *P *= 0.8552). Similarly, none of the 3-way interactions were statistically significant: larval infestation × growth habit × relative maturity (*F*_1,62_ = 0.9217, *P *= 0.3407), growth habit × relative maturity × year (*F*_1,60_ = 0.0180, *P *= 0.8936); larval infestation × growth habit × year (*F*_1,61_ = 0.0461, *P *= 0.8306), or larval infestation × relative maturity × year (*F*_1,61_ = 3.2602, *P *= 0.076).

All 2-way interactions were not significant: growth habit × relative maturity (*F*_1,60_ = 0.0404, *P *= 0.8413), larval infestation × year (*F*_1,55_ = 0.0899, *P *= 0.7654), larval infestation × growth habit (*F*_1,61_ = 0.5685, *P *= 0.4537), larval infestation × relative maturity (*F*_1,61_ = 0.3887, *P *= 0.5352), growth habit × year (*F*_1,60_ = 0.4061, *P *= 0.5263), and relative maturity × year (*F*_1,60_ = 2.9172, *P *= 0.092).

**Fig. 6.**
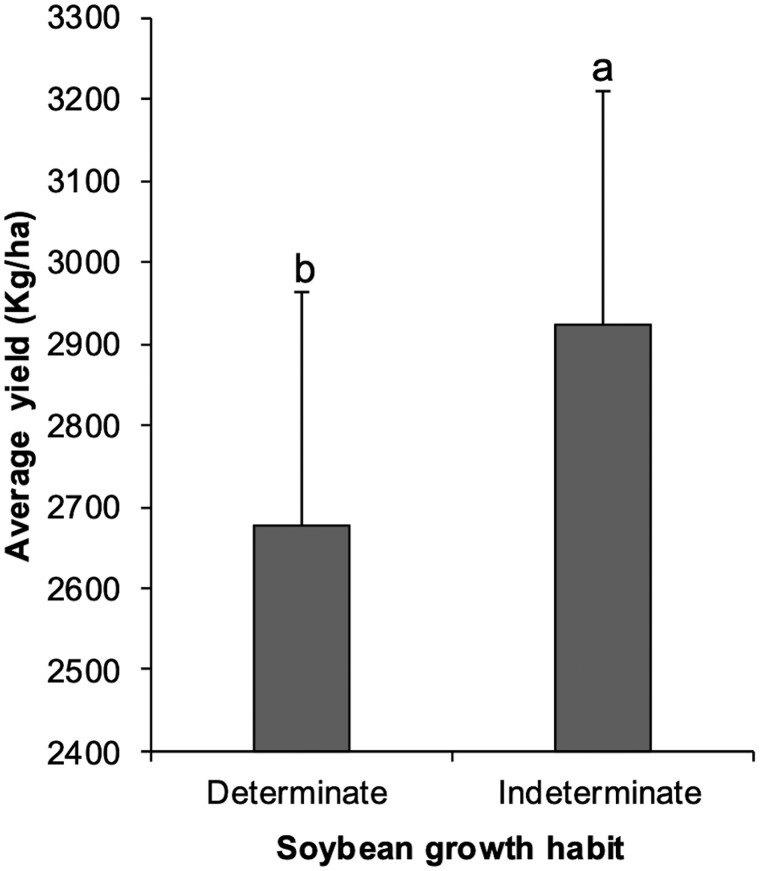
Average yield (kg/ha) for each growth habit (determinate and indeterminate) across the season. Means (±SE) followed by the same letter are not significantly different using Tukey’s HSD test at α = 0.05.

The main effects of growth habit (*F*_1,60_ = 9.3356, *P *= 0.0033) were significant (Fig. 6). While relative maturity (*F*_1,60_ = 0.7959, *P *= 0.3758), larvae infestation (*F*_1,55_ = 0.4003, *P *= 0.5295), and year (*F*_1,4_ = 0.4527, *P *= 0.5356) were not significant.

## Discussion

The overall objective of our study was to investigate if soybean varieties with different growth habits differ in their ability to compensate to *H. zea* feeding across natural infestation levels. We hypothesized that yield and yield components would not differ by growth habit (indeterminate and determinate) or relative maturity (5.2 and 5.4/5.5), regardless of *H. zea* pressure. We rejected our first hypothesis, as there was a significant relationship between larval infestation and growth habit for the yield component of individual seed weight. However, we observed no such relationship for other yield components or overall yield. We supported our second hypothesis, as relative maturity showed no relationship with larval infestation.

Seed weight tended to increase as larval infestation increased for the varieties with an indeterminate growth habit. However, this relationship was relatively weak (*R*^2^ = 0.1321). In contrast, seed weight in determinate varieties appeared neutral by infestation, which may suggest a compensatory response. We believe this relationship should be further tested in a broader range of soybean varieties, as more overlapping varieties with similar relative maturity and growth habit are now available to growers. Previous research on full-season soybean found that different growth habits exhibited similar levels of yield loss in response to *H. zea* feeding (Schug et al. 2022). Similar to our study, yield components varied widely across variety. Although Schug et al. (2022) did not observe the same effect of *H. zea* infestation on seed weight by growth habit, differences in study design and analysis may explain this discrepancy. Our approach, which treated location as a random factor, allowed us to pool data across all sites and likely provided the statistical power needed to detect this interaction.

Based on this finding, soybean with an indeterminate growth habit may exhibit greater compensatory ability in response to biotic stressors (such as *H. zea* feeding) or abiotic stressors (such as drought) than determinate growth habits. Indeterminate soybean varieties typically have longer stems and maintain reproductive tissue availability over a longer portion of the season compared to those with a determinate type (Fehr et al. 1977, Fehr and Caviness 1977, Hartung et al. 1981). This extended availability of developing tissue allows *H. zea* larvae with prolonged access to high-quality, emerging, and fully expanded trifoliates. Although larvae feed on various tissue types to fulfill nutritional gaps (Suits et al. 2017), studies show that *H. zea* egg and larvae location is similar between growth habits (Reisig et al. 2020, 2021, Possebom et al. 2024), suggesting equal susceptibility to infestation. However, the extended growth window for vegetative growth in indeterminate soybean may allow for greater compensation. It is important to note that most of our field sites experienced relatively moderate to low *H. zea* pressure, with only one site exceeding economic thresholds. These low infestation levels may have limited our ability to detect compensatory effects on yield components beyond seed weight.

The most significant finding in our study was the compensation in individual soybean seed weight following larval feeding, as growth habits differed in their mechanisms of compensating for injury and damage. However, this compensation did not translate to significant yield differences between growth habits. Moreover, we did not observe any other relationships between growth habit and relative maturity for the other yield components we measured. Therefore, when these findings are considered alongside previous research under relatively higher *H. zea* pressure in full-season plantings, Schug et al. (2022) suggest that the current economic threshold remains appropriate for both determinate and indeterminate soybean in double-crop systems.

Beyond insect pressure effects, we observed that indeterminate varieties yielded more than determinate varieties. However, since we only used 2 varieties to represent maturity, this result could be confounded by variety. For example, the 2 varieties in the 5.2 relative maturity produced more average seeds per pod than the 2 varieties in the 5.4/5.5 relative maturity. Moreover, while not significant, both the determinate varieties produced numerically more seeds and more pods per plant in 2022 than the 2 other varieties. A study incorporating a broader range of varieties within each relative maturity could help disentangle differences in yield and yield components between growth habit and across environments. However, this was beyond the scope of this study, which focused on the effects of *H. zea* feeding.

The year-to-year variations in yield performance we observed in our study suggest that environmental conditions interact with relative maturity and growth habit. Furthermore, environmental conditions likely interact with the effects of *H. zea* on yield, but these impacts may have been obscured by the inclusion of location as a random effect in our analysis. For example, Reisig et al. (2017) observed a yield impact from *H. zea* at very low infestation levels and suggested that drought conditions may have limited the plants’ ability to compensate for feeding injury. In this study, indeterminate varieties demonstrated yield compensation ability in both years. However, 2022 environmental conditions were likely more favorable for both of the determinate varieties, enabling them to maximize seed and pod production relative to other varieties. This supports the hypothesis that growth habit influences a variety’s response to specific environmental conditions, with indeterminate varieties exhibiting more consistent performance across variable environments. Furthermore, relative maturity also influences variety response as in our study 5.2 relative maturity compensated with increased seeds per plant in comparison with 5.4/5.5 relative maturity.

Our relatively low *H. zea* infestation levels might explain why we observed different outcomes than Schug et al. (2022). They found that under high infestation, 5.2 relative maturity varieties experienced greater pod injury and damage than later-maturing 5.9 varieties, primarily because early-maturing varieties with thicker pods became available during peak larval feeding periods. Had our double-crop system experienced similarly high *H. zea* pressures, our results might have aligned with these findings, potentially revealing relative maturity preferences that remained undetected under our study’s low infestation conditions. However, as we detailed earlier, there are many other differences in design and analysis between these studies.

Taken as a whole, we posit that the yield compensation differences we observed between growth habits (eg indeterminate yielding more than determinate) and maturity groups (eg 5.2 producing more seeds per pod than 5.4/5.5) may be attributed more to genetic yield potential and specific breeding goals of earlier soybean varieties rather than inherent characteristics of growth habit or relative maturity itself. This perspective is supported by extensive previous research demonstrating how soybean varieties perform differently across environmental conditions (Rincker et al. 2014), plant phenological stages (Craig et al. 1992), soil moisture retention capabilities (Williams et al. 2016), rainfall patterns (Smith and Bass 1972a, Smith and Bass 1972b; Kumudini et al. 2007; Chen and Wiatrak 2010), and timing of environmental stress or *H. zea* infestation (Supplementary Appendix 1).

Further research should be focused on sites with higher *H. zea* pressure and across more varied environments where abiotic stress may interact with direct *H. zea* herbivory. Such studies could determine the soybean plant’s compensatory responses by examining how environmental factors such as water stress, planting dates, and row spacing, as well as their interactions with *H. zea* infestation, influence yield outcomes. Additionally, further research across more varieties might better explain the relationship between seed weight and *H. zea* infestation. Understanding these factors could provide valuable insights for optimizing soybean yield and improving pest management strategies in this crop.

## Supplementary Material

toaf211_Supplementary_Data

## Data Availability

Data will be available upon request
