# Supplementary material for: Impact of Helicoverpa zea (Lepidoptera: Noctuidae) feeding on yield components in double-cropped soybean with determinate and indeterminate growth habits
Source: J Econ Entomol. 2025 Aug 23;118(5):2280–9. doi: 10.1093/jee/toaf211 (PMC12534091; doi:10.1093/jee/toaf211)
Supplement: toaf211_Supplementary_Data [file toaf211_supplementary_data.zip › Supplementary File for Review.docx]

**Appendix 1.** Average number of Helicoverpa zea larvae in 2.80 row/m in 112 plots in each year (2021 and 2022) across all locations (7 total); we sampled for larvae using a standard 0.71 m beat cloth methodology. Means (± SE) followed by the same letter are not significantly different using Tukey’s HSD test at α = 0.05.
