# Supplementary figures and images for: Impact of Helicoverpa zea (Lepidoptera: Noctuidae) feeding on yield components in double-cropped soybean with determinate and indeterminate growth habits
Source: J Econ Entomol. 2025 Aug 23;118(5):2280–9. doi: 10.1093/jee/toaf211 (PMC12534091; doi:10.1093/jee/toaf211)

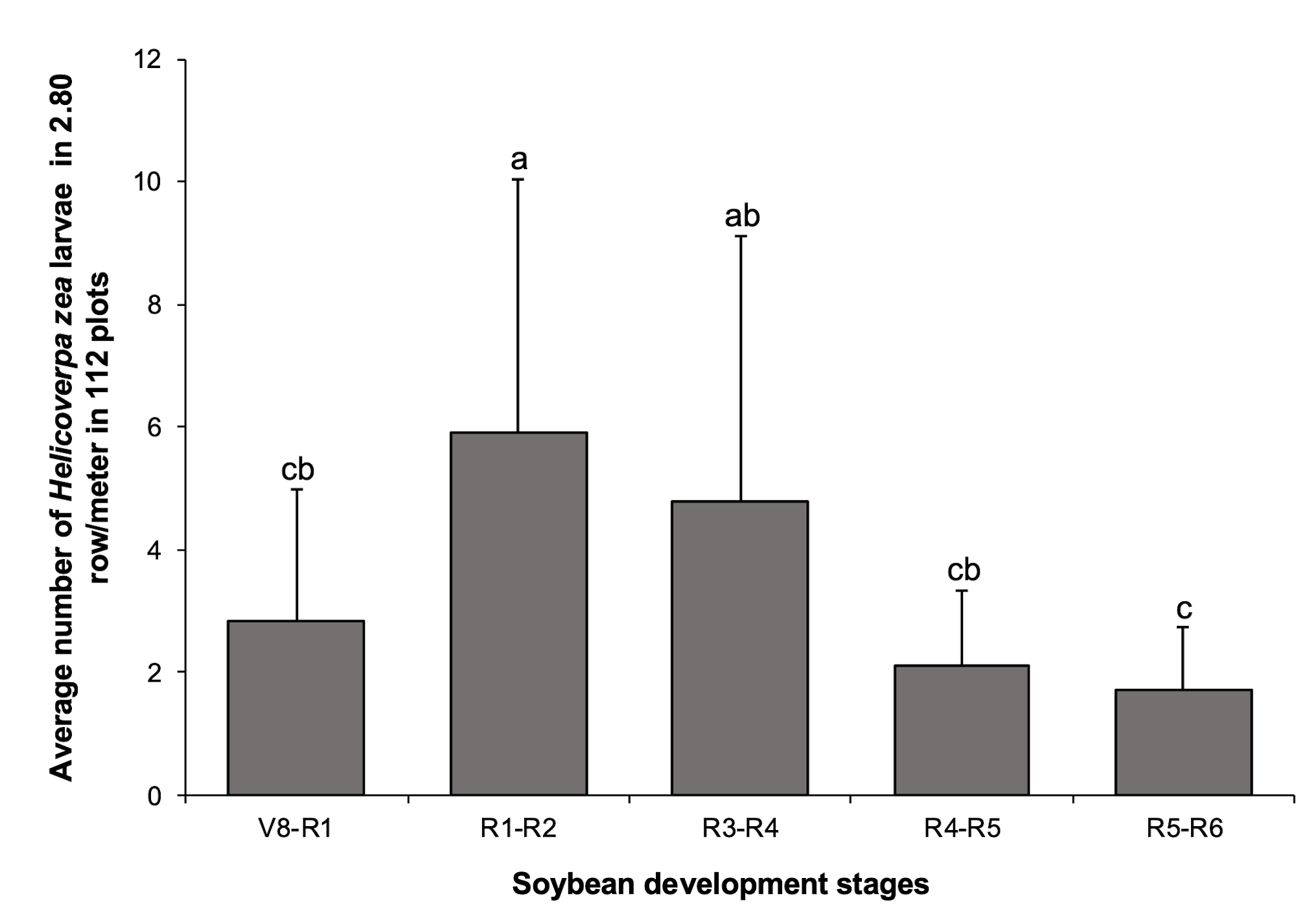

Supplement: toaf211_Supplementary_Data [file toaf211_supplementary_data.zip › Appendix 1.tiff]
